# Nodule surrounded by a halo with ground-glass opacity

**DOI:** 10.36416/1806-3756/e20250401

**Published:** 2026-03-05

**Authors:** Edson Marchiori, Bruno Hochhegger, Gláucia Zanetti

**Affiliations:** 1. Universidade Federal do Rio de Janeiro, Rio de Janeiro (RJ) Brasil.; 2. University of Florida, Gainesville (FL) USA.

**Figure 1:**
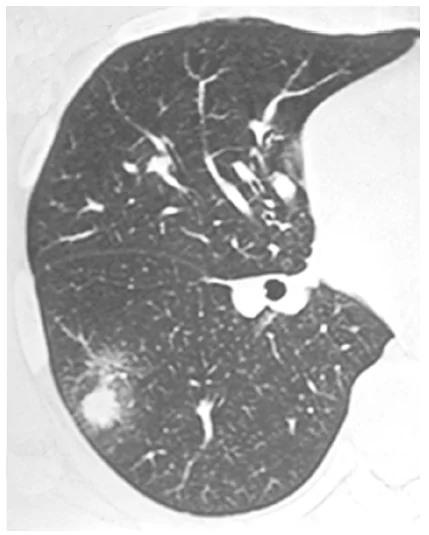
A CT scan with lung window setting at the level of the lower lobes, showing a nodule with soft tissue density in the right lower lobe, surrounded by a halo with ground-glass opacity (halo sign).

A 57-year-old female patient, smoker (70 pack-years), presented with complaints of acute respiratory infection. In the ER, a chest CT scan was ordered, which showed a solid nodule surrounded by a ground-glass opacity halo (the halo sign) in the lower lobe of the right lung (Figure 1). Initial laboratory tests were normal, including negative HIV serology, and no history of immunosuppressant use. PET/CT showed radiopharmaceutical uptake, interpreted as a malignant lesion. The patient reported frequent contact with bats in her home and had been experiencing night sweats for the past 6 months. Serology for histoplasmosis was positive.

Histoplasmosis is a fungal infection, being the most common pulmonary mycosis in the Americas. In endemic areas, pulmonary nodules are the most frequent findings, the disease generally being asymptomatic. They can vary in size, with smooth, irregular contours, or with a halo sign. The halo sign is a nonspecific tomographic finding, characterized by the presence of a halo of ground-glass opacity surrounding a nodule or, less frequently, a mass or a rounded area of consolidation.^1^ ^,^ ^2^

Useful information for the initial diagnostic approach is whether the patient is immunocompetent or immunocompromised. In immunocompromised patients, infectious causes predominate, especially angioinvasive fungal diseases. In immunocompetent patients, especially those who are asymptomatic and smokers, bronchial carcinoma, especially lepidic adenocarcinoma, should always be considered. The differential diagnosis also includes hemorrhagic metastases, lymphomas, Kaposi’s sarcoma, various types of bacterial, fungal and viral infections, as well as sarcoidosis, granulomatosis with polyangiitis (Wegener’s granulomatosis) and organizing pneumonia.^1^ ^,^ ^2^

One important aspect that deserves consideration is the limited value of PET/CT for the differential diagnosis between malignant and benign nodules. Studies show that in endemic regions for granulomatous diseases (especially tuberculosis and histoplasmosis), the specificity of PET/CT for the diagnosis of lung cancer is about 40%, since not only malignant processes but also inflammatory/infectious processes (false-positives) can have high uptake of the radiopharmaceutical. In addition, some malignant tumors, such as carcinoid and mucinous adenocarcinoma, may not uptake it (false-negatives).^3^

Thus, although the halo sign is a nonspecific finding with a broad differential diagnosis, correlation with clinical data and laboratory tests, as well as associated tomographic findings, can significantly narrow down the diagnosis, remembering that in some cases the final diagnosis is histopathological. In the presented case herein, the history of exposure to bats suggested fungal lung disease, confirmed by serology.
